# Molecular detection of *human papillomavirus-16* among Sudanese patients diagnosed with squamous cell carcinoma and salivary gland carcinoma

**DOI:** 10.1186/s13104-021-05471-5

**Published:** 2021-02-09

**Authors:** Fatima E. Mohamed, Leena N. Aldayem, Maisa A. Hemaida, Omayma Siddig, Zeinab H. Osman, Irene R. Shafig, Mohamed A. M. Salih, Mohamed S. Muneer, Rowa Hassan, Eiman Siddig Ahmed, Lamis Ahmed Hassan, Osama El Hadi Bakheet, Ali M. M. Edris, Ayman Ahmed, Nouh S. Mohamed, Emmanuel E. Siddig

**Affiliations:** 1 Department of Histopathology and Cytology, Faculty of Medical Laboratory Sciences, University of Khartoum, Khartoum, Sudan; 2 Department of Oral Pathology, Faculty of Dentistry, University of Khartoum, Khartoum, Sudan; 3 Department of Microbiology, Faculty of Medical Laboratory Sciences, Sudan International University, Khartoum, Sudan; 4 Department of Oral Pathology, Faculty of Dentistry, Ibn Sina University, Khartoum, Sudan; 5 Department of Parasitology and Medical Entomology, Faculty of Medical Laboratory Sciences, Nile University, Khartoum, Sudan; 6 Department of Clinical Chemistry, Faculty of Medical Laboratory Sciences, Karary University, Khartoum, Sudan; 7 Department of Neurology, Mayo Clinic, Jacksonville, FL USA; 8 Department of Radiology, Mayo Clinic, Jacksonville, FL USA; 9 Faculty of Medicine, University of Khartoum, Khartoum, Sudan; 10 Mycetoma Research Center, University of Khartoum, Khartoum, Sudan; 11 Department of Histopathology and Cytology, Faculty of Applied Medical Sciences, University of Bisha, Bisha, Kingdom of Saudi Arabia; 12 Institute of Endemic Diseases, University of Khartoum, Khartoum, Sudan; 13 Faculty of Medicine, Nile University, Khartoum, Sudan

**Keywords:** Human papilloma virus, Squamous cell carcinomas, Sudan

## Abstract

**Objective:**

*Human papillomavirus* (HPV) gained momentum as a potential etiological factor for many types of cancers. Therefore, the aim of this study was to assess the prevalence of HPV-16 infection among Sudanese patients diagnosed with Squamous Cell Carcinoma (SCC) and Salivary Gland Carcinoma. A descriptive, hospital-based study was conducted. 150 formalin-fixed paraffin-embedded blocks were collected.

**Results:**

The study population included a total of 150 patients aged between 18 to 87 years with a mean age of 48.8 ± 11.9 years. Based on gender, females constituted 46.7% while males constituted 53.3%. The 150 patients were classified into 40 (26.0%) esophageal, 30 (20.0%) nasopharyngeal, 18 (12.0%) conjunctival, 18 (12.0%) tongue 12 (8.0%) laryngeal, 8 (5.3%) lip, 6 (4.0%) oropharyngeal, 6 (4.0%) mucoepidermoid, and 6 (4.0%) adenoid cystic, and 6 (4.0%) myoepithelial carcinomas. Odds ratio for male and female diagnosed with carcinoma was 1.025 [0.439–2.394, 95% CI]. Molecular detection of HPV-16 revealed a prevalence of 26 (17.3%) patients were positive for HPV-16. According to cancer diagnosis, esophageal SCC patients showed a high proportion of HPV-16; 14/40 (35.0%). A statistically significant difference was seen for the distribution of HPV-16 positive patients based on cancer diagnosis, P value 0.001.

## Introduction

Human papilloma virus (HPV) encompass a group of double-stranded DNA icosahedral viruses that belongs to the family papillomaviridae with a tendency to infect mucosal and cutaneous epithelia [1]. More than 100 types of HPV have been fully identified by genome sequencing and many of them are identified as the etiology of benign and malignant tumours such as skin papillomas, cervical carcinoma and oropharyngeal carcinoma [2]. Not all HPV types have the same ability to cause tumours. High risk types of HPV are designated carcinogenic and include types 16 and 18 while low risk types are designated as probably carcinogenic and include types 6 and 11 [3]. High risk HPV are responsible for one third of virus-induced cancers which account for about 5% of human cancers [2]. Among the high risk subgroup, HPV-16 is the most potent type and is implicated in carcinogenesis of different body sites [4].

Prevalence of infection by HPV-16 varies across populations, genders and habit attributes with incident infection as high as 60% been detected [5]. The immune system is usually capable of clearing the virus in 6–18 months following an infection. However, in latent infections the double-stranded HPV genome integrates with that of the host. The E6 and E7 viral genes encode the two major oncoproteins E6 and E7, respectively, which transform the cell cycle and help the virus evade the immune system [6]. The E7-induced degradation of the tumour suppressor RB protein renders infected cells irresponsive to growth control mechanisms by promoting them to the S phase. In contrast, biological activity of E6 oncoprotein is its ability to induce the degradation of p53 tumour suppressor gene via ubiquitation pathway [7]. Our knowledge about the involvement of HPV-16 in various human cancers has led to significant advances in prevention and management these cancers. For instance, the HPV vaccine decreased the burden of cervical cancer and other HPV-associated diseases, and identification of the subset of oropharyngeal carcinoma that is HPV-16 positive showed a unique impact especially in terms of treatment and prognosis [8, 9]. Therefore, in this study we aimed at investigating the prevalence of HPV-16 among Sudanese patients diagnosed with mucosa carcinomas.

## Main text

### Materials and methods

#### Study design and samples

A descriptive, cross-sectional study was conducted from January 2018 till June 2019. Formalin-fixed paraffin-embedded (FFPE) blocks of cases diagnosed with a variety of carcinomas were retrieved from the archive of the Radiation and Isotopes Centre Khartoum regardless of their sex and ages. Sections from the cases that were stained by hematoxylin and eosin were then reviewed to confirm the diagnosis.

#### Ethical clearance

The study ethics approval and consent to participate were obtained by the ethics review board of the Faculty of Medical Laboratory Sciences, University of Khartoum. Informed consent was obtained from each participant prior to enrollment using writing informed consent.

#### DNA Extraction from The Paraffin Embedded Formalin-Fixed Blocks

Since HPV-16 cannot easily be cultured, nowadays the most reliable method used in HPV-16 detection is detection of its DNA by Polymerase Chain Reaction (PCR) [10]. Sections of 25 μm were cut in triplicates from each FFPE block, and transferred into 2 ml Eppendorf tubes. To dissolve the paraffin wax, 1 ml of xylene was added, incubated for 30 min, and centrifuged at 13,000 rpm for 5 min. This process was repeated in order to ensure complete removal of wax. Supernatants were discarded and rehydration of the precipitate was made using a series of ethanol concentrations starting from absolute ethanol, followed by 95%, through 90%, and lastly into 70% ethanol, for 3 min in each ethanol concentration. After the last 70% ethanol wash, residual ethanol was let to evaporate via incubation at 37 °C for 15 min. Dry wax-free tissue sections were then prepared for DNA extraction using QIAamp DNA FFPE Tissue Kit, according to the manufacturer instructions (Qiagen, Germany). DNA quality were checked using nanodrop spectrophotometer (Implen, Germany). Extracted DNA was stored at -20 C for subsequent molecular investigation.

#### Molecular detection of HPV-16

Molecular detection of HPV-16 was made using the previously published primers; forward primer 5-TTT TGG GTT ACA CAT TTA CAA G-3 and the reverse primer 5-TGT CTG CTT TTA TAC TAA CCG-3 for the PCR amplification [11]. PCR reaction mixture was made on a PCR single-tube ready to use iTaq PCR pre-mix according to manufacturer instructions (iNtRON Biotechnology Inc, South Korea). PCR amplification process was started with initial denaturation at 95 °C for 5 min, followed by 40 cycles of denaturation step at 95 °C for 30 s, annealing step at 55 °C for 30 s, and extension step at 72 °C for 1 min. And finally, an extension step for 10 min at 72 °C.

PCR amplicons were visualized using UV-transilluminator (Bio.Doc-it UVP, Cambridge, UK), after loaded into 2.5% agarose gel (iNtRON biotechnology, South Korea) and electrophoresed at 100 V for 60 min (Bio-RAD Brand, USA). To avoid false negative and false positive results, PCR results were recorded as positive results in comparison to a 100 bp molecular marker (iNtRON biotechnology, South Korea) based on the presence of 121 bp band size in all the three DNA samples for each FFPE block. A double distilled water was used as negative control in each PCR run instead of adding DNA.

### Statistical analysis

Data were analysed using the Statistical Package for Social Sciences software (SPSS, v 20.0). Chi-Squared test was used to compare the status of HPV-16 infection among the study variables. Odds ratio was calculated with 95% Confidence Interval (CI). A *P* value < 0.05 was considered statistically significant.

### Results

The study population included a total of 150 participants aged between 18 to 87 years with a mean age of 48.8 ± 11.9 years. Based on gender, females constituted 46.7% while males were constituted 53.3% of our cohort. Odds ratio for male and female diagnosed previously with cancer was 1.025 [0.439–2.394, 95% CI]. The cohort included 40 patients diagnosed with esophageal squamous cell carcinoma (SCC), 30 patients with nasopharyngeal carcinoma, 12 patients with laryngeal carcinoma and 6 patients with oropharyngeal carcinoma. Eighteen cases in our cohort had SCC of the conjunctiva, 18 patients had tongue SCC and 8 patients had SCC of the lip. Interestingly, 18 of the patients had carcinomas of the salivary gland carcinoma split equally between mucoepidermoid carcinoma, adenoid cystic carcinoma and myoepithelial carcinoma.

Based on age group, the age group of 31–60 years constituted the majority of the study population 80.0% (120/150), of them 54/120 (45.0%) were females and 66/120 (55.0%) were males. A statistically significant difference was noted for the distribution of gender and cancer diagnosis based on age groups, P values 0.01 and 0.004, respectively (Table 1).

**Table 1:** The distribution of gender, cancer diagnosis, and the prevalence of HPV-16 based on age groups

	Age group			Total	P value
	1–30	31–60	61–90		
Gender					
Female	0 (0.0%)	54 (77.1%)	16 (22.9%)	70 (46.7%)	0.010
Male	6 (7.5%)	66 (82.5%)	8 (10.0%)	80 (53.3%)	
Cancer diagnosis					
Esophageal SCC	0 (0.0%)	32 (80.0%)	8 (20.0%)	40 (26.7%)	0.004
Nasopharyngeal carcinoma	0 (0.0%)	22 (73.3%)	8 (26.7%)	30 (20.0%)	
Tongue	2 (11.1%)	16 (88.9%)	0 (0.0%)	18 (12.0%)	
Conjunctiva SCC	0 (0.0%)	16 (88.9%)	2 (11.1%)	18 (12.0%)	
Mucoepidermoid carcinoma	0 (0.0%)	6 (100%)	0 (0.0%)	6 (4.0%)	
Adenoid cystic carcinoma	0 (0.0%)	6 (100%)	0 (0.0%)	6 (4.0%)	
Myoepithelial carcinoma	2 (33.3%)	2 (33.3%)	2 (33.3%)	6 (4.0%)	
Lips SCC	0 (0.0%)	6 (75.0%)	2 (25.0%)	8 (5.3%)	
Larynx	2 (16.7%)	8 (66.7%)	2 (16.7%)	12 (8.0%)	
Oropharyngeal carcinoma	0 (0.0%)	6 (100%)	0 (0.0%)	6 (4.0%)	
HPV-16					
Negative	2 (1.6%)	104 (83.9%)	18 (14.5%)	124 (82.7%)	0.002
Positive	4 (15.4%)	16 (61.5%)	6 (23.1%)	26 (17.3%)	
Total	6 (4.0%)	120 (80.0%)	24 (16.0%)	150 (100%)	

**Fig. 1:**
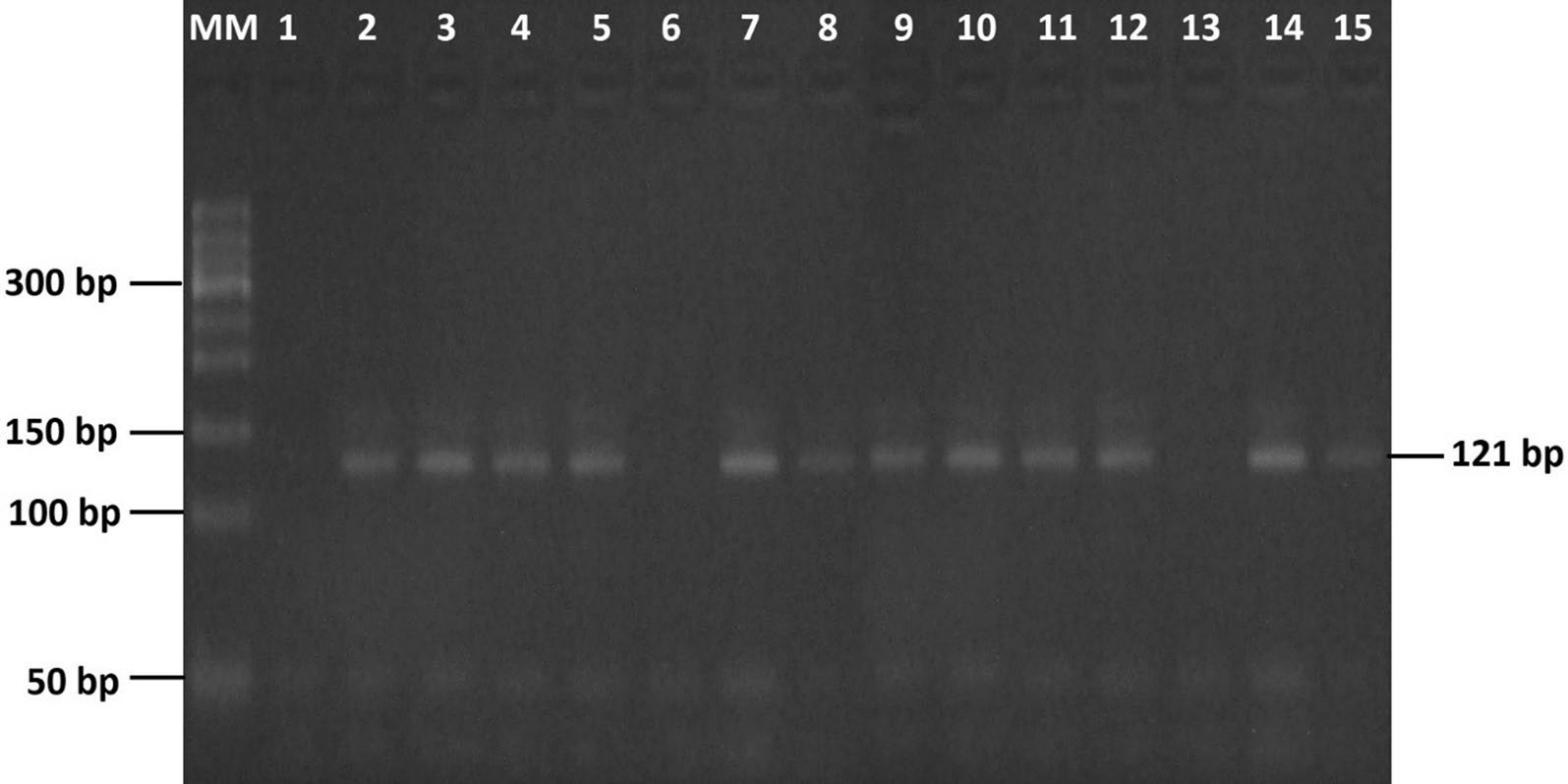
PCR amplification of HPV-16. MM: molecular marker of 50 bp length. 1: Negative control, 2: Positive control, 3–5, 7–12, 14 and 15: represent HPV-16 positive samples. 6 and 13: Negative samples for the presence of HPV-16

The molecular detection revealed that 17.3% of the study samples were positive for HPV-16 based on the presence of 121 bp long PCR amplicons (Fig. 1).

We did not find association between the status of infection by HPV-16 and gender as 17.5% and 17.1% of the males and females, respectively, were positive for HPV-16, P value 0.564. HPV-16 positive cases were distributed according to age into 16 (13.3%) were in the age group of 31–60 years, followed by 6 (25.0%) and 4 (66.7%) of the age groups 61–90 years and 1–30 years, respectively. The prevalence of HPV-16 among the study population was statistically significant based on age group, P value 0.002. According to cancer diagnosis, those were diagnosed as esophageal SCC showed a high proportion of HPV-16 positive patients; 14/40 (35.0%), followed by 6/12 (50.0%), 4/18 (22.2%), and 2/6 (33.3%) of those diagnosed with larynx cancer, tongue cancer, and adenoid cystic adenocarcinoma, respectively. A statistically significant difference was seen for the distribution of HPV-16 positive patients based on cancer diagnosis, P value 0.001. The distribution of HPV-16 positive patients based on gender, age groups, and cancer diagnosis is illustrated in Table 2.

**Table 2:** The distribution of HPV-16 positive patients based on gender, age groups, and cancer diagnosis

	HPV-16		Total	P value
	Negative	Positive		
Gender				
Female	58 (82.9%)	12 (17.1%)	70 (46.7%)	0.564
Male	66 (82.5%)	14 (17.5%)	80 (53.3%)	
Age group				
1–30 years	2 (33.3%)	4 (66.7%)	6 (4.0%)	0.002
31–60 years	104 (86.7%)	16 (13.3%)	120 (80.0%)	
61–90 years	18 (75.0%)	6 (25.0%)	24 (16.0%)	
Cancer diagnosis				
Esophageal SCC	26 (65.0%)	14 (35.0%)	40 (26.7%)	0.001
Nasopharyngeal carcinoma	30 (100%)	0 (0.0%)	30 (20.0%)	
Tongue	14 (77.8%)	4 (22.2%)	18 (12.0%)	
Conjunctiva SCC	18 (100%)	0 (0.0%)	18 (12.0%)	
Mucoepidermoid carcinoma	6 (100%)	0 (0.0%)	6 (4.0%)	
Adenoid cystic carcinoma	4 (66.7%)	2 (33.3%)	6 (4.0%)	
Myoepithelial carcinoma	6 (100%)	0 (0.0%)	6 (4.0%)	
Lips SCC	8 (100%)	0 (0.0%)	8 (5.3%)	
Larynx	6 (50.0%)	6 (50.0%)	12 (8.0%)	
Oropharyngeal carcinoma	6 (4.0%)	0 (0.0%)	6 (4.0%)	
Total	124 (82.7%)	26 (17.3%)	150 (100%)	

### Discussion

The involvement of HPV-16 in various human cancers has led to significant advances in prevention and management of these cancers. For instance, the identification of the subset of oropharyngeal carcinoma that is HPV-16 positive showed unique impacts in the treatment and the prognosis of cancer [12, 13]. Therefore, in this study we aimed at investigating the prevalence of HPV-16 among Sudanese patients diagnosed with different types of cancers. Interestingly, the association between status of HPV-16 infection and the type of tumor that this study has found reveals many areas that would improve management of patients in Sudan. The prevalence of HPV-16 infection in esophageal SCC that we found in our study was higher than what was estimated by several meta-analyses reporting averages of 11.4% and 18.5% [14, 15]. Although an etiologic relationship between HPV-16 and esophageal SCC has not been concluded yet, a meta-analysis suggested that patients with HPV-16 positive esophageal SCC are more likely to have an improved survival than patients with HPV-16 negative SCC [16]. When lip and tongue SCC are combined and considered as oral SCC, we found a low prevalence of HPV-16 infection in our cohort. Although HPV-16 has long been identified as risk factor for oral cancer [17], its contribution is lower oral cancer than in oropharyngeal cancer [18]. A finding that is worth highlight is that none of the cases diagnosed with oropharyngeal carcinoma were positive for HPV-16, in contrast to previous studies such as the series from the United Kingdom which found a prevalence of 70% [18]. Although the world now is moving towards de-escalating the treatment strategies for patients with oropharyngeal carcinoma because of its better response and associated survival [19], it seems too early for this in Sudan. Although a study in the United Kingdom identified a subset of nasopharyngeal carcinoma that is associated with HPV-16 [20], none of our cases showed HPV-16 positivity. This is in line with the established role of Epstein-Barr Virus (EBV) rather than HPV in the etiology of this cancer [21], and with previous studies in Sudan that showed a high prevalence of EBV in nasopharyngeal carcinoma [22]. The equal distribution of HPV-16 positive and HPV-16 negative laryngeal carcinoma is higher than the 25% that was estimated by a meta-analysis on the prevalence of HPV-16 in this carcinoma [23]. This highlights the need for routine testing of this cancer for HPV-16, especially a recent meta-analysis showed that patients with HPV-16 positive laryngeal carcinoma have better survival [24]. The low prevalence of HPV-16 in the salivary gland carcinoma in this study support previous studies in that HPV-16 is not associated with these tumors [25].

### Conclusion

This study concludes that those who were diagnosed as esophageal SCC show a high proportion of HPV-16 and is worth mentioning that none of the cases diagnosed with oropharyngeal carcinoma were positive for HPV-16 which indicate a negative role for HPV-16 for oropharyngeal carcinoma initiation. Also, this study highlights the need for a routine testing of HPV-16.

## Limitation

- Samples investigated in this study were limited, therefore a larger sample size and incorporate different types of head and neck cancers could provide further insight.
- Studying the relation between the presence of HPV-16 and prognosis of the cancer to correlate the disease progression with the presence and absence of HPV-16 were not applicable to be done.

## Data Availability

The datasets used and/or analyzed during the current study are available from the corresponding author on reasonable request.

## Abbreviations

EBV: Epstein-Barr Virus
FFPE: Formalin-fixed paraffin-embedded
HPV: Human papilloma virus
SCC: Squamous cell carcinoma
